# Inheritance

**Published:** 1894-11-10

**Authors:** 


### INHERITANCE.

Great interest attaches to certain questions regarding mixed heredity raised by Sir James Paget in his address to the Abernethian Society. It was long thought that the fœtus *in utero* could only be infected from the mother; the semen may, however, convey disease directly to the ovum, and there is reason to believe that the mother may become infected through the fœtus. If then the child can receive an impress of disease from either side, what happens, asks Sir James Paget, when such diseases as cancer and tuberculosis, which do not appear to thrive together in the same individual, are mingled together by inheritance? Additional interest has been added to this matter by the researches of Maffucci, whose paper in the *Annals of Surgery*, October, 1894, on "Infective Embryonal Pathology" is likely to throw a good deal of light on this subject. Desiring to know if embryonal tissue had the same power of resistance to a virus as the tissues of the adult, he inoculated various microbes into hen's eggs, and at different periods of the brood period, inoculated also the albumen enclosing the embryo. He found that although the fecundated and brooded albumen was favourable to the growth of microbes, it did not induce their growth in the living embryo, nor did microbes that were present in the tissues of the embryo multiply. The embryo may contain many microbes which will kill the control animal, but it remains unaffected by them, and the microbes can be attenuated or destroyed in the tissues of the embryo, but not in the surrounding albumen.

Maffucci next inoculated buck conies with mammalian tuberculosis and examined the semen. Tubercle bacilli were difficult to detect in the first few hours, but they were found to be present in great numbers from the twenty-fifth day up to three months; and sea-hogs inoculated with this semen became tubercular. Other buck conies inoculated in the same way with tuberculosis were put with does which became pregnant. It was quite exceptional for sea-hogs inoculated with the organs of a fœtus of a tubercular father to become tubercular, and rabbits by a tubercular father did not show tubercular lesions until two months after birth.

The fœtuses of mothers grown tubercular during pregnancy can contain the bacillus four hours after the inoculation of the mother, and the organs of these fœtuses rendered sea-hogs tubercular in the first forty-eight hours, and most of the sea-hogs not rendered tubercular in this way died of marasmus as if they had been inoculated with dead bacilli. It thus appears that during its life the embryo can resist the growth of pathogenic microbes in its tissues, and can destroy or attenuate them, and perhaps accumulate them and develop them later in life.

